# Supplementary material for: Prospective Evaluation of Ustekinumab Dose Intensification in Inflammatory Bowel Diseases
Source: Gastro Hep Adv. 2026 Jul 9;5(10):101054. doi: 10.1016/j.gastha.2026.101054 (PMC13475195; doi:10.1016/j.gastha.2026.101054)
Supplement: Supplementary file 1 [file mmc1.pdf]

Supplementary Materials

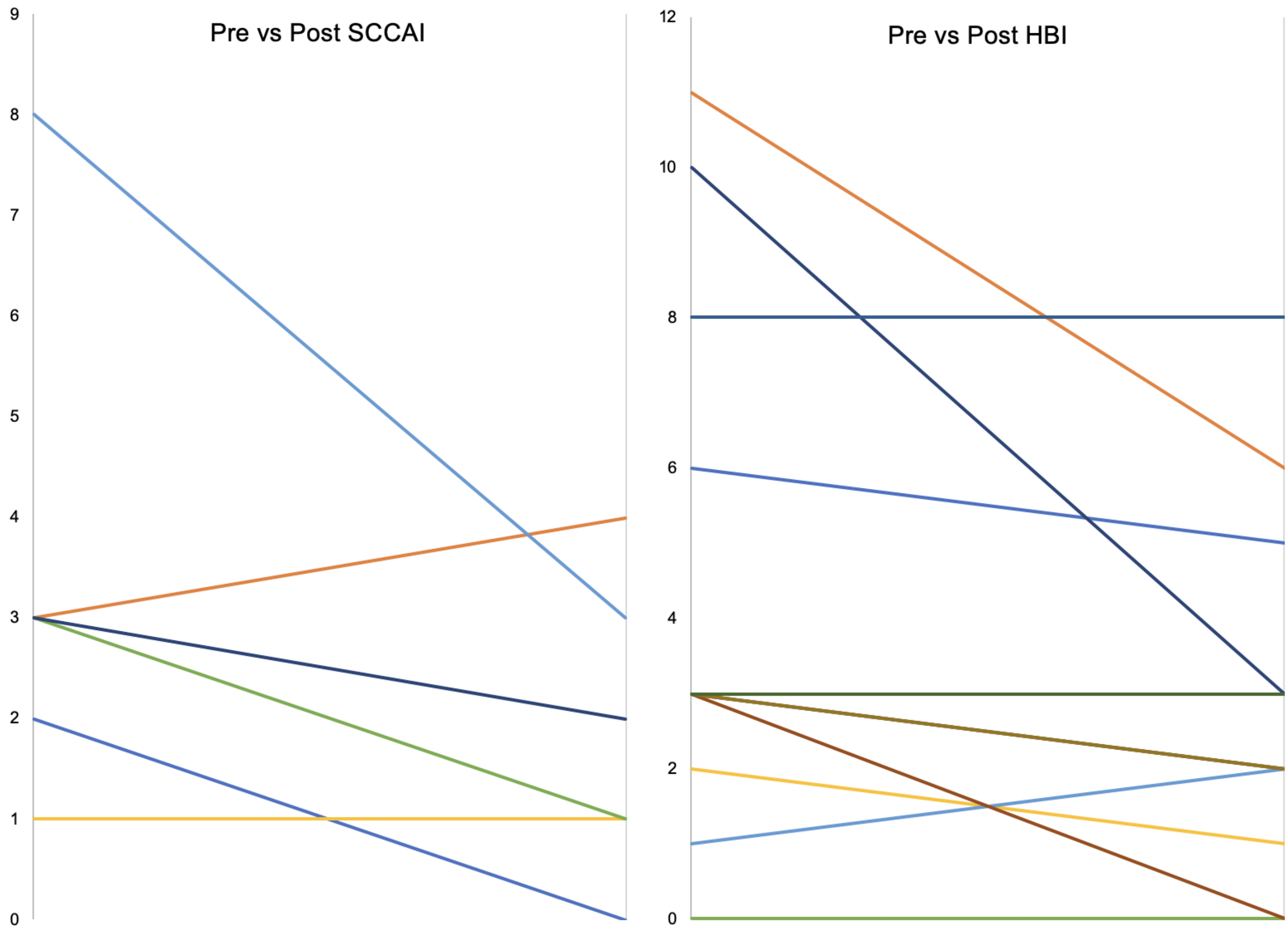

Supplementary Figure 1. Paired line plots of pre vs post-ustekinumab dose intensification disease activity scores (after maintenance dose 3).
Abbreviations: SCCAI-simple clinical colitis activity index, HBI=Harvey-Bradshaw Index
