# Supplementary material for: Prospective Evaluation of Ustekinumab Dose Intensification in Inflammatory Bowel Diseases
Source: Gastro Hep Adv. 2026 Jul 9;5(10):101054. doi: 10.1016/j.gastha.2026.101054 (PMC13475195; doi:10.1016/j.gastha.2026.101054)
Supplement: Supplementary file 2 [file mmc2.pdf]

Gastro Hep Advances 2026;5:101054

ORIGINAL RESEARCH—CLINICAL

Prospective Evaluation of Ustekinumab Dose Intensification in Inflammatory Bowel Diseases

Rahul S. Dalal, Heidy J. Cabral, and Jessica R. Allegretti

Division of Gastroenterology, Hepatology and Endoscopy, Department of Medicine, Brigham and Women's Hospital, Harvard Medical School, Boston, Massachusetts

BACKGROUNDS AND AIMS: Ustekinumab is an effective therapy for moderate-to-severe inflammatory bowel diseases (IBD), including Crohn's disease (CD) and ulcerative colitis (UC). However, secondary loss of response to standard every-8-week maintenance dosing is common. Prospective data supporting dose intensification are limited. To prospectively evaluate the effectiveness and safety of ustekinumab dose intensification to every 4 weeks (q4w) or every 6 weeks (q6w) in patients with IBD experiencing secondary loss of response. **METHODS:** We conducted a prospective cohort study of adults with CD or UC undergoing empiric ustekinumab dose intensification to q4w or q6w. Clinical disease activity was assessed using the Harvey-Bradshaw Index for CD and the Simple Clinical Colitis Activity Index for UC at baseline and following intensified maintenance dosing. The primary outcome was steroid-free clinical remission after the third intensified dose. Secondary outcomes included clinical response, biochemical response using fecal calprotectin, changes in serum ustekinumab concentrations, and adverse events. **RESULTS:** Nineteen patients (12 CD, 7 UC) were enrolled. After the third intensified dose, steroid-free clinical remission was achieved in 75.0% of CD and 71.4% of UC patients, while over 80% of patients achieved clinical response. Outcomes were similar between q4w and q6w regimens. Serum ustekinumab concentrations increased significantly following dose intensification, whereas biochemical response rates were modest. No new safety signals were observed, and no patients discontinued therapy due to adverse events. **CONCLUSION:** Ustekinumab dose intensification to q4w or q6w was safe and associated with improved clinical outcomes in this prospective IBD cohort.

Keywords: Ulcerative Colitis; Crohn's; Inflammatory Bowel Disease; Biologics; Dose Optimization

Introduction

Inflammatory bowel diseases (IBD), including Crohn's disease (CD) and ulcerative colitis (UC), are lifelong autoimmune conditions of the gastrointestinal tract. Intestinal inflammation associated with these disorders can lead to severe diarrhea, abdominal pain, and decreased quality of life. Over the last decade, advanced therapies targeting various immune system pathways have emerged, with a goal of inducing clinical and endoscopic remission. Due to variability in durable treatment responses, many patients undergo dose optimization of their treatment regimens.

Ustekinumab is an interleukin-12 and 23 antagonist that is approved for the treatment of moderate-to-severe IBD. Standard therapy includes a weight-based infusion followed by subcutaneous maintenance injections every 8 weeks (q8w). Clinical trial data indicate that this regimen is effective in inducing clinical remission in 53% of CD patients and 44% of UC patients by week 44 after induction.[1,2]

Real-world evidence suggests that up to 50% of individuals with IBD have loss of clinical response to ustekinumab, and many undergo empiric dose intensification to every 4 weeks (q4w) or every 6 weeks (q6w).[3] Several cohort studies in CD[3–6] and UC[7,8] suggest that dose intensification is effective in recapturing clinical response to ustekinumab. However, these studies are limited by their retrospective design and variability in post-intensification clinical evaluation for response. We therefore performed a prospective evaluation of ustekinumab dose intensification to guide clinical decision-making after patients exhibit secondary loss of response to q8w maintenance dosing.

Methods

This was a prospective cohort study of adults prescribed ustekinumab for the treatment of CD or UC undergoing empiric dose intensification to either q4w or q6w after loss of clinical response to q8w dosing (at the discretion of the primary prescribing physician). Patients were included if they had active IBD symptoms (Harvey-Bradshaw Index [HBI] >4 points for CD or Simple Clinical Colitis Activity Index [SCCAI] >2 points for UC) or required IV or oral corticosteroids while receiving standard dosing of ustekinumab. Patients with disease activity scores below these thresholds must have been

Abbreviations used in this paper: **AE, adverse event; CD, Crohn's disease; HBI, Harvey-Bradshaw Index; IBD, inflammatory bowel disease; q4w, every 4 weeks; q6w, every 6 weeks; q8w, every 8 weeks; SCCAI, Simple Clinical Colitis Activity Index; SFCR, steroid-free clinical remission; UC, ulcerative colitis.**

Most current article

© 2026 The Authors. Published by Elsevier Inc. on behalf of American Gastroenterological Association Institute. This is an open access article under the CC BY license (http://creativecommons.org/licenses/by/4.0/).
2772-5723
https://doi.org/10.1016/j.gastha.2026.101054

requiring IV or oral corticosteroids at the time of enrollment. Exclusion criteria were prior total proctocolectomy, ustekinumab prescribed for non-IBD indications, pouchitis diagnosis, combination therapy with another advanced therapy, concurrent cytotoxic chemotherapy or immune checkpoint inhibitor therapy, or initiation of an immunomodulator (azathioprine, 6-mercaptopurine, or methotrexate) within 1 week of dose intensification. Patients were followed for up to 20 weeks post-dose intensification.

Patients were interviewed at baseline (within 6 weeks prior to first intensified dose at q4w or q6w) for HBI/SCCAI scores. Patients were also interviewed by phone at 7 to 10 days after intensified maintenance doses 1, 2, and 3 for HBI/SCCAI scores as well as medication changes and adverse events (AEs). Patients were asked to submit stool tests for fecal calprotectin and blood tests for serum ustekinumab concentrations at baseline (within 10 days prior to first intensified dose) and at follow-up (ustekinumab concentrations within 7 days prior to the week 16 dose for q4w patients and within 7 days prior to the week 18 dose for q6w patients; stool calprotectins during week 16 for both groups). Additionally, medical charts were reviewed for the following data: demographics, age of IBD diagnosis, prior/concurrent IBD medications (including prednisone or oral budesonide), cigarette smoking, prior intestinal surgeries, gastroenterology clinic notes within the last 12 months for examination findings such as perianal abscess or fistulae, and occurrence of an IBD-related hospitalization within the last 12 months.

The primary outcome was steroid-free clinical remission (SFCR; HBI $<$ 5 points or SCCAI $<$ 3 points with no use of corticosteroids) at 7 to 10 days after intensified maintenance dose 3. Secondary outcomes included clinical response (decline in SCCAI or HBI by $\geq$ 3 points from the pre-dose intensification measurement) after maintenance dose 3 and biochemical response (either normalization of follow-up fecal calprotectin [<50 mcg/g] or reduction by 50%). Other outcomes were SFCR/clinical response at 7 to 10 days after intensified maintenance doses 1 and 2 and AEs. Descriptive statistics were used to present results, which were stratified by IBD type and q4w vs q6w regimens for the primary and secondary outcomes. Fisher's exact test was used to compare proportions. Paired *t*-tests were used to compare ustekinumab concentrations and fecal calprotectin levels at baseline and follow-up. Analyses were exploratory and not controlled for multiplicity.

Results

We enrolled 19 patients, 12 with CD and 7 with UC between 2/1/2023 and 7/1/2025. The mean age was 41.1 years; 12 (63.1%) patients were female, 8 (42.1%) patients were dose intensified to q4w, and 11 (57.9%) patients were dose intensified to q6w. The mean baseline SCCAI was 3.0 and HBI was 4.4. Two patients (10.5%) were receiving oral corticosteroids at baseline. Other baseline characteristics are presented in Table 1.

After intensified maintenance dose 3, 75.0% of CD and 71.4% of UC patients achieved SFCR ($P = 1.0$), 83.3% of CD and 85.7% of UC achieved clinical response ($P = 1.0$), and 44.4% of CD and 20.0% of UC achieved biochemical response ($P = .58$) (Figure 1). When stratifying by maintenance regimen, 62.5% q4w and 81.8% q6w achieved SFCR ($P = .60$), 75.0% q4w and 90.9% q6w achieved

Table 1. Baseline Characteristics

Characteristic	Every 4 wk (n = 8)	Every 6 wk (n = 11)
Age, mean (SD), y	45.8 (19.7)	40.6 (17.3)
Sex	4/8 (50.0%)	8/11 (72.7%)
Race		
Caucasian	7/8 (87.5%)	10/11 (90.9%)
Unknown/declined	1/8 (12.5%)	1/11 (9.1%)
Hispanic	2/8 (25.0%)	1/11 (9.1%)
Current smoker	0	0
Baseline disease activity		
SCCAI	2.5 (0.7)	3.8 (4.1)
HBI	3.2 (2.9)	5 (3.2)
Prior advanced therapy exposures		
None (ie, bio-naïve)	0	3/11 (27.2%)
Infliximab	3/8 (37.5%)	6/11 (54.5%)
Adalimumab	5/8 (62.5%)	5/11 (45.5%)
Certolizumab	0	1/11 (9.1%)
Vedolizumab	2/8 (25.0%)	4/11 (36.4%)
Risankizumab	0	0
Janus kinase inhibitor	1/8 (12.5%)	2/11 (18.2%)
Current oral/IV corticosteroids	1/8 (12.5%)	1/11 (9.1%)
Current immunomodulator	1/8 (12.5%)	0
Perianal disease	2/8 (25.0%)	0
IBD hospitalization within 12 mo	1/8 (12.5%)	1/11 (9.1%)
Prior IBD surgery	1/8 (12.5%)	1/11 (9.1%)
Stool calprotectin, mean (SD), mcg/g	879 (990)	117 (142)

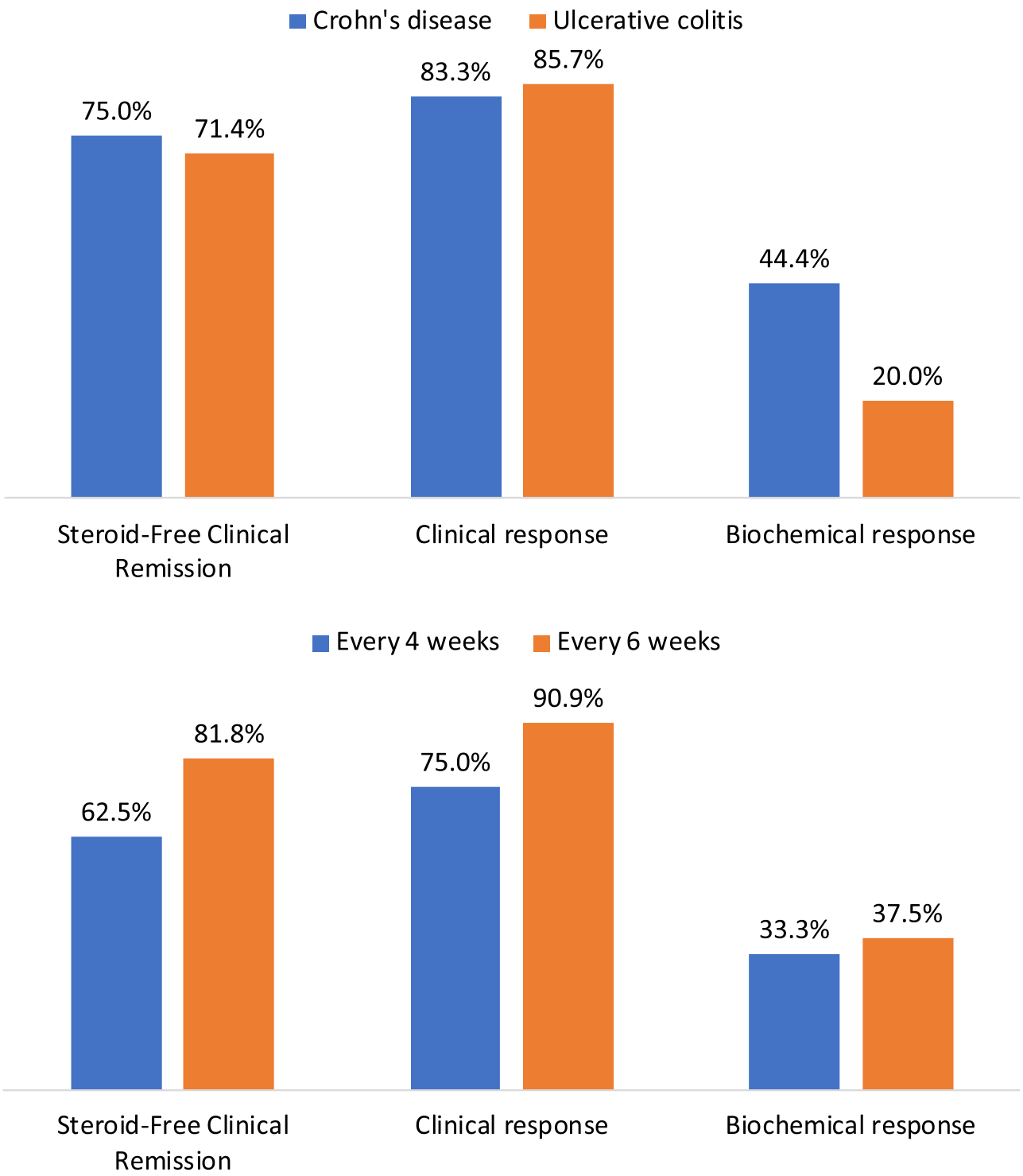

Figure 1. Primary and secondary outcomes stratified by IBD type and intensified dosing regimen.

clinical response ($P = .55$), and 33.4% q4w and 37.5% q6w achieved biochemical response ($P = 1.0$) (Figure 1). The mean baseline ustekinumab concentration was 4.9 mcg/mL, which increased to 6.7 mcg/mL ($P = .002$) at follow-up. The mean baseline fecal calprotectin was 450.6 mcg/g, which was 437.3 at follow-up ($P = .39$). Paired line plots of disease activity scores pre- vs post-dose intensification are presented in Supplementary Figure 1.

After intensified maintenance doses 1 and 2, rates of SFCR for the overall cohort were 73.6% and 78.9%, respectively. For clinical response, they were 84.2% and 84.2%, respectively.

Nine (47.3%) patients reported 14 AEs during follow-up. Serious AEs (n = 2) included an emergency room visit for shortness of breath and weakness and a hospitalization for appendicitis and COVID; however, neither was attributed to ustekinumab. Other AEs included increased abdominal cramping, bloating, cold sore, upper respiratory tract infection, fatigue (n = 3), back pain, headaches, arthralgia, and increased urgency. No patients discontinued therapy due to an AE.

Discussion

In this prospective cohort study, ustekinumab dose intensification to q4w or q6w was associated with high rates of SFCR and clinical response among patients with either CD or UC who experienced loss of response to standard maintenance dosing. By the third intensified maintenance dose, over 70% of patients in both disease subtypes achieved SFCR and >80% achieved clinical response. No significant differences were observed between CD and UC or between intensified dosing regimens. These findings provide important prospective evidence supporting ustekinumab dose intensification as a viable management strategy across IBD phenotypes.

Notably, we observed similarly high rates of SFCR and response with both q4w and q6w regimens. Although numerically higher SFCR and response rates were observed in the q6w group, these differences were not statistically significant, likely reflecting limited sample size rather than true equivalence or superiority. Additionally, patients in the q4w group may have had more refractory disease given the higher mean baseline fecal calprotectin, confounding these results. Nevertheless, these findings suggest that q6w dosing may represent a clinically meaningful intermediate intensification strategy for selected patients, potentially balancing efficacy, patient burden, and payer considerations.

Despite robust clinical improvements, biochemical response rates were modest, and mean fecal calprotectin levels did not significantly decline over the follow-up period. This may be partly due to the inclusion of patients treated with corticosteroids at baseline, which suppresses inflammation, as well as the relatively low baseline fecal calprotectin level in the q6w group. The discordance between clinical and biochemical outcomes has been reported previously with ustekinumab and other biologics and may reflect delayed mucosal healing, persistent subclinical inflammation, or limited sensitivity of fecal calprotectin in certain disease phenotypes. Importantly, serum ustekinumab concentrations increased significantly following dose intensification, supporting the pharmacokinetic rationale for this strategy and suggesting that inadequate drug exposure contributes to secondary loss of response in a subset of patients. Longer follow-up may be required to determine whether sustained dose intensification translates into improved biochemical or endoscopic outcomes.

Empiric dose intensification was well-tolerated in this cohort, with no new safety signals identified. Nearly half of patients reported at least one AE during follow-up, the majority of which were mild and self-limited. These findings are consistent with prior real-world studies demonstrating the safety of ustekinumab dose intensification.

Our results corroborate and extend prior retrospective real-world studies demonstrating the effectiveness of ustekinumab dose intensification in IBD.[8,9] Previous cohorts have reported clinical response rates ranging from 60% to 85% following intensification to q4w dosing, with somewhat variable remission rates depending on definitions and follow-up duration. However, much of the existing literature is limited by retrospective designs, heterogeneous timing of outcome assessments, and reliance on chart-based symptom documentation. The present study addresses several of these limitations by prospectively capturing standardized symptom indices at predefined intervals after each intensified dose, allowing for a

precise assessment of early therapeutic benefit following dose intensification.

Several limitations warrant consideration. The modest sample size limited statistical power to detect differences between disease subtypes and dosing regimens, and the follow-up duration may have been insufficient to assess biochemical or endoscopic response. Dose intensification strategy was determined by treating clinicians rather than randomized assignment, introducing potential selection bias and confounding by indication for the q4w group. Additionally, endoscopic outcomes were not systematically assessed.

Conclusion

Ustekinumab dose intensification to q4w or q6w appears to be a safe and potentially effective strategy to improve clinical outcomes in IBD patients with secondary loss of response to standard dosing. Larger, prospective, and randomized studies with long-term follow-up are warranted to further refine dose optimization strategies.

Supplementary Materials

Material associated with this article can be found, in the online version, at https://doi.org/10.1016/j.gastha.2026.101054.

References

1. Sandborn WJ, Gasink C, Gao LL, et al. Ustekinumab induction and maintenance therapy in refractory Crohn's disease. N Engl J Med 2012;367(16):1519–1528.
2. Sands BE, Sandborn WJ, Panaccione R, et al. Ustekinumab as induction and maintenance therapy for ulcerative colitis. N Engl J Med 2019;381(13):1201–1214.
3. Dalal RS, Njie C, Marcus J, et al. Predictors of ustekinumab failure in Crohn's disease after dose intensification. Inflamm Bowel Dis 2020;27(8):1294–1301.
4. Ollech JE, Normatov I, Peleg N, et al. Effectiveness of ustekinumab dose escalation in patients with Crohn's disease. Clin Gastroenterol Hepatol 2020.
5. Kopylov U, Hanzel J, Liefferinckx C, et al. Effectiveness of ustekinumab dose escalation in Crohn's disease patients with insufficient response to standard-dose subcutaneous maintenance therapy. Aliment Pharmacol Ther 2020;52(1):135–142.
6. Fumery M, Peyrin-Biroulet L, Nancey S, et al. Effectiveness and safety of ustekinumab intensification at 90 Mg every four weeks in Crohn's disease: a multicenter study. J Crohns Colitis 2020.
7. Dalal RS, Esckilsen S, Barnes EL, et al. Predictors and outcomes of ustekinumab dose intensification in ulcerative colitis: a multicenter cohort study. Clin Gastroenterol Hepatol 2021.
8. Yang H, Li B, Guo Q, et al. Systematic review with meta-analysis: loss of response and requirement of ustekinumab dose escalation in inflammatory bowel diseases. Aliment Pharmacol Ther 2022;55(7):764–777.
9. Dalal RS, Pruce JC, Allegretti JR. Long-term outcomes after ustekinumab dose intensification for inflammatory bowel diseases. Inflamm Bowel Dis 2023;29(5):830–833.

Received April 16, 2026. Accepted July 6, 2026.

Correspondence:
Address correspondence to: Rahul S. Dalal, Crohn's and Colitis Center, Brigham and Women's Hospital, 850 Boylston Street, Suite 201, Chestnut Hill, Massachusetts 02467. e-mail: rsdalal@bwh.harvard.edu.

Authors' Contributions:
Rahul S. Dalal: Study concept and design, acquisition of data, statistical analysis, analysis and interpretation of data, drafting of manuscript. Heidy J. Cabral: Acquisition of data. Jessica R. Allegretti: Study concept and design, analysis and interpretation of data, critical revision of the manuscript for important intellectual content, study supervision.

Conflicts of Interest:
These authors disclose the following: Rahul S. Dalal has served as a consultant for Centaur labs, Janssen, and Takeda and has received grant support from Pfizer and Janssen. Jessica R. Allegretti serves as a consultant for Janssen, Pfizer, AbbVie, Seres Therapeutics, Ferring, GSK, Merck, Bristol Myers Squibb, Roivant, and Adiso; serves as a speaker for BMS, AbbVie, and Janssen; and has received research support from Pfizer, Janssen, and Merck. The remaining author discloses no conflicts.

Funding:
This work was funded by Johnson & Johnson.

Ethical Statement:
This study was approved by the institutional review board of Brigham and Women's Hospital (Boston, MA).

Data Transparency Statement:
Due to the small sample size and to protect patient health information, data and study materials will not be made available to other researchers. Analytical methods are provided in the main manuscript text.

Reporting Guidelines:
This manuscript followed the STROBE guidelines for the reporting of cohort studies.

Gastro Hep Advances, Volume 5

Supplemental information

Prospective Evaluation of Ustekinumab Dose Intensification in Inflammatory Bowel Diseases

Rahul S. Dalal, Heidy J. Cabral, and Jessica R. Allegretti

Supplementary Materials

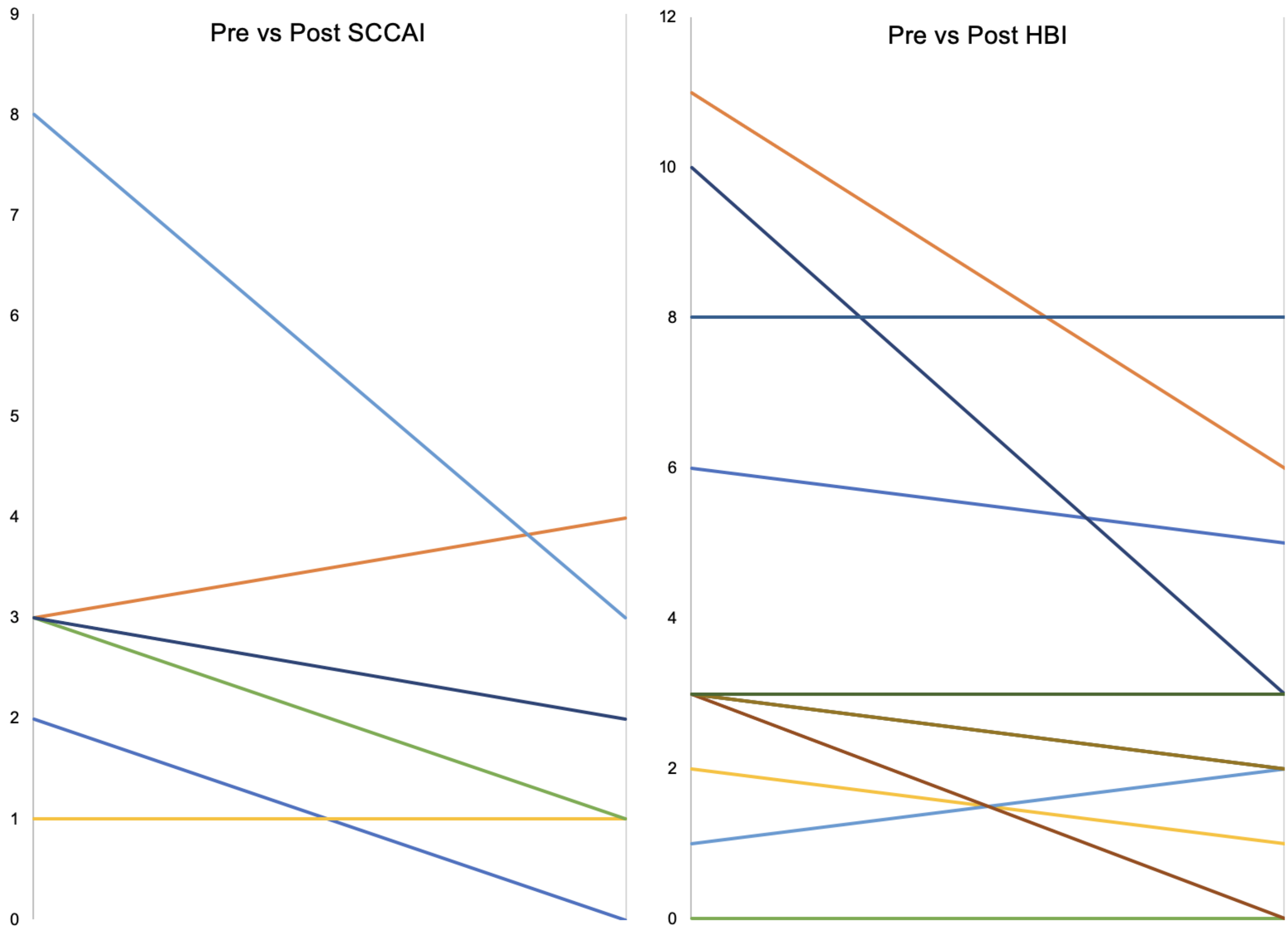

Supplementary Figure 1. Paired line plots of pre vs post-ustekinumab dose intensification disease activity scores (after maintenance dose 3).
Abbreviations: SCCAI-simple clinical colitis activity index, HBI=Harvey-Bradshaw Index
